# Dengue fever diagnosis in resource-limited settings

**DOI:** 10.1017/S0950268825100460

**Published:** 2025-08-22

**Authors:** Zuleihat Eneyamire Baje, Nafiu Lawal, Muhammad Bashir Bello, Mustapha Umar Imam

**Affiliations:** 1 Centre for Advanced Medical Research and Training, Usmanu Danfodiyo University Sokoto, Sokoto, Nigeria; 2 Department of Veterinary Microbiology, Faculty of Veterinary Medicine, Usmanu Danfodiyo University Sokoto, Sokoto, Nigeria; 3 Infectious Disease Research Department, King Abdullah International Medical Research Center, Riyadh, Saudi Arabia; 4 Department of Medical Biochemistry, College of Health Sciences, Usmanu Danfodiyo University Sokoto, Sokoto, Nigeria

**Keywords:** dengue, diagnosis, fever, mosquitoborne illnesses, PCR, RDT, tourniquet test

## Abstract

Dengue is an arboviral infection that poses a substantial public health concern, with early diagnosis being a critical factor in effective management. However, limited diagnostic expertise in developing countries contributes to the under-reporting of dengue cases. This review compares the accuracy of rapid diagnostic tests (RDTs) and the tourniquet test (TT) in diagnosing dengue fever (DF) in non-laboratory-based settings. Relevant original articles on the use of RDTs and TT for dengue diagnosis were retrieved from PubMed, Scopus, and ScienceDirect. The STARD and QUADAS-2 tools were employed to evaluate the methodological quality of the included studies. Search terms included combinations of ‘fever’, ‘dengue’, and ‘“diagnosis’. In total, 23 articles were eligible for inclusion. The RDTs demonstrated mean sensitivities and specificities of 76.2% (SD = 13.8) and 91.5% (SD = 10.3), respectively, while the TT showed mean sensitivity and specificity values of 48.6% (SD = 24.9) and 79.5% (SD = 14.9), respectively. Overall, RDTs exhibited superior diagnostic performance compared to the TT. Our findings suggest that the TT is an inadequate stand-alone diagnostic tool for dengue. RDTs should be prioritized for dengue diagnosis in resource-limited settings. However, in situations where RDTs are unavailable, the TT may serve as a supplementary option.

## Introduction

With increasing population growth, urbanization, and industrialization have collectively contributed to widespread circulation of dengue virus (DENV), which is primarily transmitted by *Aedes* species mosquitoes [1, 2]. An estimated 390 million people are infected annually, making DENV a major health concern due to recurrent outbreaks of dengue fever (DF) and dengue haemorrhagic fever (DENF). DENV is a single-stranded, positive-sense RNA-enveloped virus with an approximately 11 kb genome that encodes three structural proteins (envelop, capsid, and membrane) and seven non-structural proteins (NS1-NS5) [2, 3].

While most DENV infections are asymptomatic, approximately 5–10% of cases can progress to severe forms such as dengue hemorrahgic fever (DHF) or dengue shock syndrome (DSS). These life-threatening complications are primarily driven by vascular fragility, resulting from endothelial dysfunction and a cytokine storm initiated by the host immune system. NS1 protein plays a critical role in this pathology by directly damaging endothelial cells through complement system activation and induction of pro-inflammatory cytokines, including tumour necrosis factor alpha (TNF-α), interleukin-6 (IL-6), and interferon gamma (IFN-γ). These cytokines, released by monocytes, dendritic cells, and T cells contribute to excessive complement activation, leading to vascular leakage, pleural effusion, and plasma extravasation. The resulting loss of endothelial integrity can lead to hypotension, haemoconcentration, and, in severe cases, hypovolemic shock [2, 4].

Early diagnosis of DENV is essential for proper management and public health response to the disease [5]. Dengue is primarily confirmed via laboratory testing using various techniques, including viral isolation, molecular assays, and serological methods [6]. While virus isolation is regarded as the gold standard, it is laborious, time-consuming, and requires biosafety level 3 facilities. Reverse transcription-polymerase chain reaction (RT-PCR) and enzyme-linked immunosorbent assay (ELISA) are more commonly used to detect viral RNA or specific antibodies [7]. Nevertheless, these methods still require trained personnel and specialized machines, which are often lacking in laboratories in resource-limited settings.

This diagnostic gap has led to the increasing use of rapid diagnostic tests (RDTs), often called lateral-flow-based point-of-care tests (Figure 2). These assays detect DENV antigens or antibodies (e.g. NS1, IgM, or IgG), and are particularly suitable for low-resource settings because of their affordability, user friendly, and minimal storage requirements [8]. RDTs can differentiate between primary and secondary dengue infections. In primary infections, IgM is the first antibody to appear, followed by IgG. In contrast, secondary infections are characterized by an early and pronounced IgG response due to immunological memory. NS1 antigen, which is produced early in infection and independent of the host’s immune response, can be detected in both primary and secondary infections, making it a valuable early marker [9].

Aside from RDTs, the tourniquet test (TT), sometimes called capillary fragility test, is a low-cost, rapid physical examination method for the diagnosis and classification of DF. Historically, the World Health Organization (WHO) discouraged its use for diagnosing DHF and DSS. However, revised WHO guidelines now include TT as a diagnostic criterion for DF, dengue with warning signs, and severe dengue, recognizing its practical utility in resource-limited settings [10, 11].

DENV infection increases capillary permeability, a phenomenon exploited by the TT. As part of the procedure, a blood pressure cuff is inflated on the upper arm to a level between systolic and diastolic pressures. After five minutes, the number of petechiae (small, non-raised, purplish-red skin dots caused by capillary haemorrhages) within a defined one-square-inch area is counted. A result of more than 20 petechiae per square inch is considered positive [12], although some guidelines also consider 10 petechiae or more to indicate a positive result [13].

Clinical features such as fever and leukopaenia can only identify ‘probable dengue’ in endemic areas. However, when combined with a positive TT (indicative of haemorrhagic manifestations), these features significantly improve diagnostic specificity in distinguishing DF from other febrile illnesses, particularly in adults [14]. For paediatric patients, the 1997 WHO clinical case definition for probable DF remains applicable: fever accompanied by a positive TT and leukopaenia during non-epidemic periods or fever with either a positive TT or leukopaenia along with any other clinical symptom during an epidemic [15].

Given the simplicity and affordability of the TT and the growing availability of RDTs in resource-constrained countries, this study aims to compare their diagnostic accuracy for DF. As infectious diseases continue to emerge and re-emerge at unprecedented rates, particularly in low-resource settings, the availability of simple, cost-effective diagnostic tools like TT and RDTs could prove life saving.

## Method

### Search strategy

A systematic search was carried out using online bibliographic databases: PubMed, Science Direct, and Scopus. The population, intervention, comparator, and outcome (PICO) question format was used for the search terms. People infected with DENV were included in the population. RDTs (to identify DENV NS1 and antibodies) and a TT (to diagnose dengue) were used as the intervention, while a validated laboratory-based PCR served as comparison. The primary outcome was DENV infection, which was assessed in studies by (1) NS1, IgM, and IgG detection for RDTs, (2) counting the number of petechiae after inflating a blood pressure cuff on a person’s upper arm for TT. A petechiae count of 10 or more indicates a positive test, indicating sensitivity, whereas a count of less than 10 indicates a negative test, indicating specificity.

**Figure 1.**
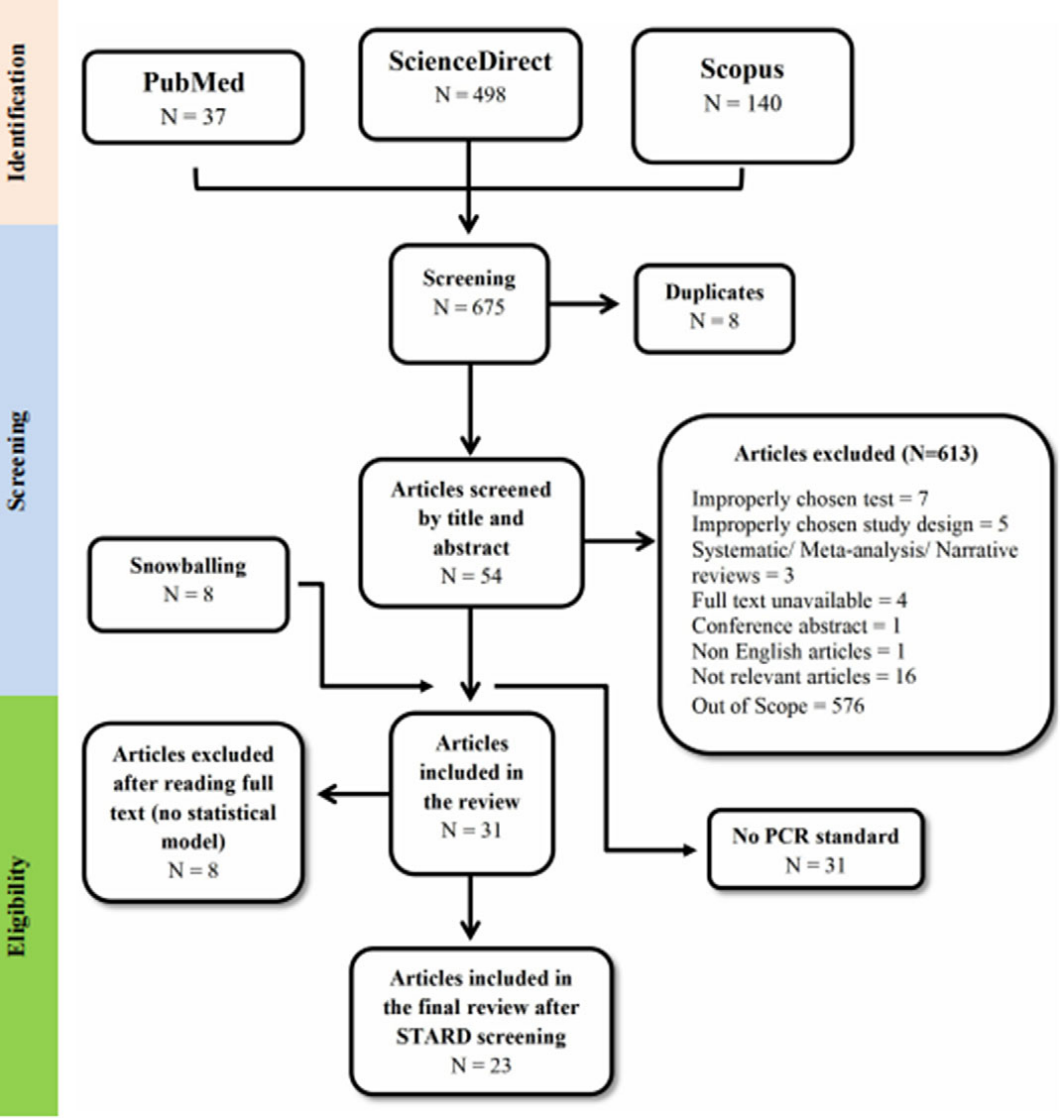
Flowchart detailing the study selection procedure. STARD = Standards for the Reporting of Diagnostic Accuracy Studies; N = number of papers.

Search terms (free-text terms and keywords) related to dengue diagnosis were utilized in the right combination with relevant controlled vocabulary (Medical Subject Heading-MeSH), along with Boolean operators to achieve the most comprehensive search results. Each search strategy was refined to improve article relevance and comprehensiveness. The final search was performed using the following terms: ‘tourniquet test’, ‘capillary fragility test’, ‘PCR’, ‘polymerase chain reaction’, ‘rapid diagnostic test’, ‘dengue’, ‘break-bone fever’, ‘diagnosis’, and ‘ELISA’ (see Supplementary Table 1). We looked for additional references in the reference lists of all the included studies that were included (i.e. snowballing) (see Figure 1 below). Only articles written in English were analyzed. Studies were considered if they satisfied the criteria described below: (a) diagnosed DF from any country of the world; (b) contained data from all types of observational research (such as cross-sectional, case report, case-control, cohort studies, and case series) that assessed the TT’s and/or RDT’s diagnostic accuracy for dengue infection; (c) studies examining individuals who initially presented with fever and were later tested for dengue using the index test, TT, or RDTs (testing for the presence of viral antibodies (IgM and/or IgG) or NS1 antigen) and PCR (reference standard), (d) studies using serum samples, plasma, or whole blood (fresh or frozen) from patients clinically suspected of dengue infection exposure.

Studies that failed to meet the research topic or inclusion criteria (not connected to the diagnosis of DF, no report on the accuracy, sensitivity, or specificity of the type of test, and/or no data to calculate it) were excluded. Studies having an undetermined methodology, challenge studies, experimental research, etc., were disregarded. Conference abstracts, brief papers with incomplete datasets or presentations, review papers, pieces containing commentary or opinion, protocols, and inaccessible articles were disqualified.

### Study selection

All the cohort studies that were found in the databases were evaluated separately by two reviewers (ZB and MBB). Potential studies were divided into groups for full-text reading. Any differences were settled by (NF) and (MUI), and the justifications for including and omitting trials were noted.

### Extraction of data and quality assessment

Data were extracted from the full texts of selected articles. The review of full text was done by ZB and MBB. Using this form, we were able to gather data on the study’s design, the types of participants, the index and reference tests, and the total number of participants. For each research study comparing the two tests, a 2x2 table was made. The quality of the data extraction table was developed following the Standards for Reporting of Diagnostic Accuracy Studies (STARD) [16] and Quality Assessment of Diagnostic Accuracy Studies-2 (QUADAS-2) [17] guidelines. The STARD each has 5 items consisting of background and objective, methods, results, discussion, and registration, and the QUADAS tools also have 4 items each consisting of patient selection, index test, reference standard, flow, and timing sections of the result. Risk of bias and applicability items are included for each of the domains mentioned above. Items were given a score of positive (low-bias risk), negative (high-bias risk), or unclear (insufficient information). The findings section included a description of each evaluation. Furthermore, the preferred reporting items for systematic reviews and meta-analyses (preferred reporting items for systematic reviews and meta-analyses (PRISMA)) were followed [18].

### Data synthesis and analysis

A 2x2 contingency table was created for each investigation. We calculated likelihood ratios (LRs), predictive values, sensitivity, and specificity. The value of 1 was added to cells in the 2x2 table of TT in the primary research that had 0 in them so that computations could be performed; however, this only occurred in one trial. Although we had intended to exclude primary papers that reported two cells with 0, that did not happen. Each study’s sensitivity and specificity were combined, and the mean output and standard deviation (SD) were estimated.

## Result

### Study selection

Out of the 675 studies initially identified, eight were excluded as duplicates (Figure 1). The remaining 667 articles were screened based on their titles and abstracts. Of these, 613 were not included for reasons listed below: out of scope (n = 576), systematic/meta-analysis/narrative reviews (n = 3), full text unavailable (n = 4), conference abstracts (n = 1), non-English language (n = 1), not relevant to the research question (n = 16), use of inappropriate diagnostic tests (n = 7), unsuitable study design (n = 5), lack of sufficient statistical data to determine true/false positives (n = 8), and absence of PCR as the reference standard (n = 31).

**Figure 2.**
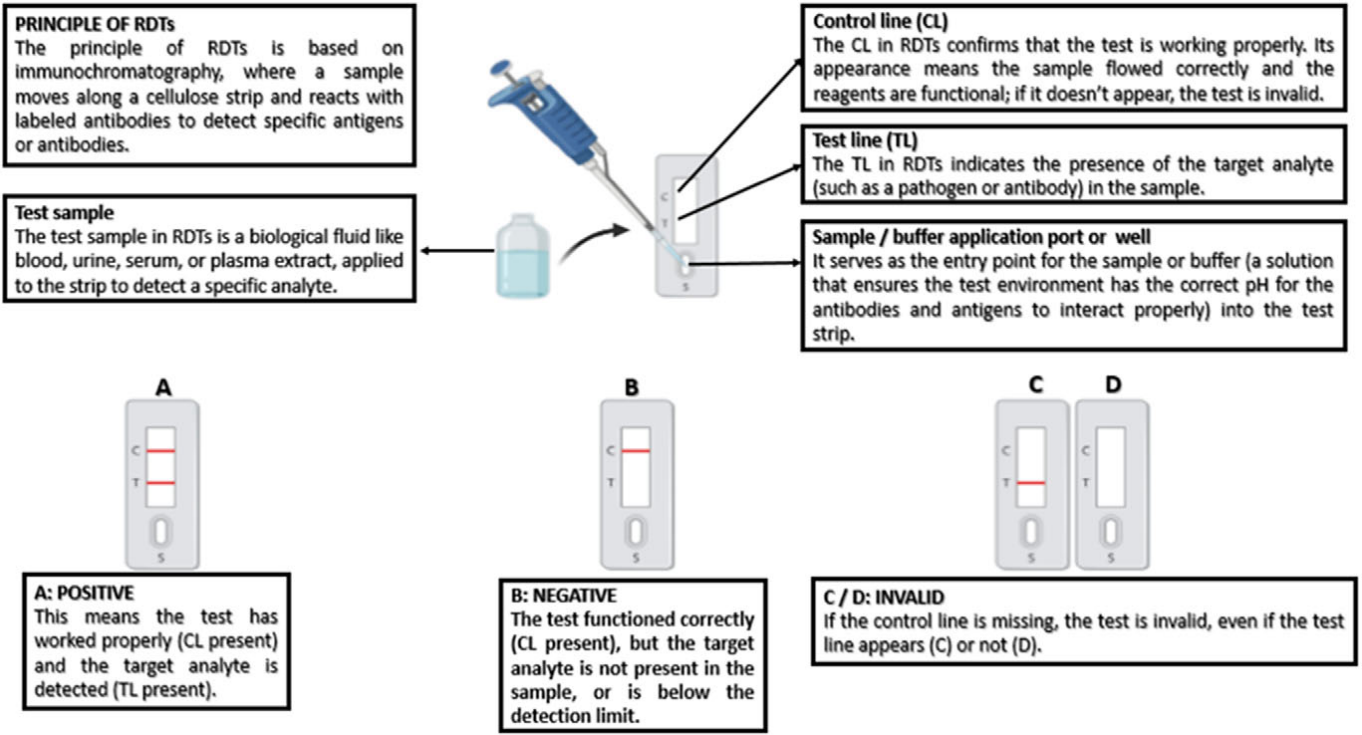
A diagram illustrating the working principle of Rapid Diagnostic Tests (RDTs).

Ultimately, 23 original research articles met the inclusion criteria and provided information on the sensitivity and specificity of dengue RDTs and the TT compared to PCR-based diagnosis. Of these, 18 studies evaluated RDTs and 5 focused on the TT. Four were prospective cohort studies, while the remaining 19 were retrospective cohort studies (Table 1). The number of participants per study ranged from 67 to 30,760.

**Table 1.** Shows the Different Studies Evaluating the Performance of Dengue RDTs and TT Using PCR as a Reference Standard.

RDTs RESULT												
Author/ Country	Test(s) Evaluated	No of samples tested using RDT	Study design/Sample type	Patient characteristics	Reference method	Sensitivity/95%CI	Specificity/95%CI	PPV/95%CI	NPV/95%CI	PLR	NLR	Comments
[3] Republic of Korea	Dengue NS1 Rapid Test	142	Retrospective/ serum	100 sera from healthy donors in Korea, along with 42 dengue NS1 antigen–positive sera from Malaysia (15), Brazil (17), and India (10), were included in the study. confirmed dengue cases, of all ages.	RDT kit (SD BioLine Dengue NS1 Ag from Alere Inc)	92.9%	100%	100%	92.9%	0%	0.1%	A highly accurate dengue NS1 rapid test is developed using anti-DENV NS1 mAbs. The results indicate that the RDT kit developed in this study is more excellent to detect a dengue NS1 antigen.
[19] Australia.	RDTs Platelia NS1 Antigen Assay	822	Retrospective/ blood or urine	The study retrospectively included all adult and paediatric patients’ samples from the Victorian Infectious Diseases Reference Laboratory, Australian state of Victoria who underwent dengue serology testing.	PCR	96.4% (92.3 – 98. 7)	98.4% (94.5 – 99.8%)	98.8% (95.3 – 99.7)	95.5% (90.5 – 97.9)	60.25%	0.04	The performance of the Platelia Dengue NS1 Antigen EIA test in the cohort of travelers who have returned from their trip satisfies the requirement as a single diagnostic test for acute dengue infection.
[20] New Caledonia	RDTs Biosynex® Dengue NS1 Assay	472	Retrospective/ serum	Patients were individuals presenting with dengue-like illness in northern New Caledonia, sampled within 7 days of symptom onset, whose specimens underwent NS1 antigen RDT at Kone Hospital and confirmatory RT-PCR at the reference laboratory between March 2017 and December 2018.	RT-PCR	79.9% (72.8 – 85.5)	96.2% (93.5 –97.9)	91.1% (85 – 95)	90.8% (87.2 –93.5)	21.03%	0.21%	A good performance was found for the Biosynex® NS1 RDT. The performance was better in very early samples (0–4 days post fever onset). In addition, the test was more sensitive in the detection of DENV–1-positive samples.
[21] Japan.	Point-of-care testing based on LAMP	67	Prospective and Retrospective/ serum or urine	The study included patients aged 16 years and above who had traveled to endemic regions and were suspected or confirmed to have dengue, Chikungunya, or Zika virus infections, with samples collected at the National Center for Global Health and Medicine, Tokyo, between 2008 and 2019 and diagnoses confirmed by RT-PCR.	RT-PCR	69.6%	81.8%	95.1%	34.6%	3.82%	0.4%	The overall concordance between the LAMP results and the RT-PCR results was 71.6%. LAMP had high sensitivity and specificity for diagnosing dengue within five days of onset.
[22] Taiwan	SD BIOLIN Dengue DUO Rapid Test Kit (NS1, IgM/ IgG).	1,607	Retrospective/ blood and serum	1,607 patients in a tertiary teaching hospital in southern Taiwan during August–September 2015, using the Taiwan Triage and Acuity Scale were enrolled. Eligible patients presented with symptoms such as fever, malaise, or post-orbital headache and resided in epidemic neighborhoods.	RT-PCR	90 – 100 % (62 – 100)	50 – 69 % (34–88)	50 – 91%/ (29–96)	80 – 100% (51–100)	1.8 – 1.4%	0.2 – 0.01%	The result suggested that the NS1-based test with or without a combination of IgM and IgG tests has good diagnostic performances in detecting dengue infections, even in the afebrile or elderly populations dengue NS1, IgM, and IgG rapid test kit was much easier to use in the clinical virological laboratory, demonstrating satisfactory sensitivity and cost-effectiveness.
	SD BIOLIN Dengue DUO Rapid Test Kit (NS1)					80 – 100 % / (62– 100)	85 %/ (91)	95% (99)	70 – 100% (49–100)			
	SD BIOLIN Dengue DUO Rapid Test Kit (IgM)					89 % (100)	75% (89)	62% (91)	50% (99)			
	SD BIOLIN Dengue DUO Rapid Test Kit (NS1/IgM)					90– 100% (62 – 100)	47–69% (11–89)	95% (30–98)	100% (51–100)			
[23] Italy	Colorimetric RDT (NSI)	373	Retrospective/ blood	From January 2014 to July 2019, patients with dengue-like symptoms in Lazio Region, Italy, were retrospectively reviewed, with samples sent to National Institute for Infectious Diseases Rome for virological confirmation.	RT-PCR	95.8% (78.9–99.9)	97.9% (94.6–99.4)	85.2% (88.5– 93.8)	99.5% (96.4–99.9)	45.62 %	0.04%	Although confirmatory tests are still necessary, our experience strongly supports the use of combined NS1 and IgM/IgG rapid dengue tests as first-line tools for prompt case identification. The use of these tests aids clinical management, surveillance activities, and vector control strategies
	Fluorimetric RDT (NS1)					84.6% (54.5–98.1)	100% (93.2–100)	100%	96.3% (87.9–98.4)	0%	0.15%	
	Colorimetric RDT NS1 and IgM					87.2% (72.6–95.7)	97.9% (95.8–99.2	82.9% (69.8–91.1)	98.5% (96.7–99.3)	41.5%	0.13%	
	Fluorimetric RDT, NS1, and IgM/IgG					96.2% (80.4–99.9)	96.2% (89.3–99.2)	89.3% (73.3–96.2)	98.7% (91.8–99.8)	25.32%	0.04%	
[24] Vietnam	Bio-Rad NS1 Antigen Strip	245	Retrospective/ plasma	The study included patients above 6 months of age with clinically suspected dengue and fever for less than 7 days, enrolled in the DENCO study at three hospitals in Ho Chi Minh City, Vietnam, between August 2006 and May 2007.	RT-PCR	61.6%/ (55.2 – 67.8)	100%/ (98.1 – 100)	100%/(93.0 – 100)	33.3% (25.6 – 41.8)	0%	0.38%	These data suggest that the NS1 test component of these assays is highly specific and has similar levels of sensitivity. The IgM parameter in the SD Duo test improved overall test sensitivity without compromising specificity (100%). The SD Dengue Duo lateral flow rapid test deserves further prospective evaluation in dengue-endemic settings.
	SD BIOLINE Dengue Duo NS1					62.4%/ (56.1 – 68.5)	100%/ (98.1 – 100)	100%/ (98.0 – 100)	33.8%/ (26.0 – 42.3)	0%	0.38%	
	SD BIOLINE Dengue Duo NS1/IgM					75.%/ (69.6 –80.8)	100%/ (93.8 – 100)	100%/ (98.4 – 100)	43.9%/ (34.3 – 53.9)	0%	0.25%	
	SD BIOLINE Dengue Duo NS1, IgM/, IgG					83%/ (78.4 –88.1)	97.9%/ (88.7 – 99.9)	99.5%/(97.3–100.0)	53.5%/ (42.4 – 64.3	39.86	0.17%	
[25] India.	NS1/IgM RDT (Dengue Day 1)	211	Cohort study/ blood sample	Enrolled children admitted to St. John’s Medical College Hospital, Bangalore, between October 2014 and October 2015 with suspected or probable dengue.	RT-PCR and sequencing	89.4% (83.9 – 93.5)	93.8% (79.2 – 99.2)	98.8% (95.6 – 99.9)	61.2% (46.2 – 74.8)	14.42%	0.11%	This NS1/IgM RDT can be a useful point-of-care assay for rapid and reliable diagnosis of acute dengue and an excellent surveillance tool in our battle against dengue since it showed high sensitivity throughout the acute phase of illness, in primary and secondary infections, in the different severity groups, and detected all the four serotypes, including co-infections.
	NS1					82.7% (76.3 – 87.9)	96.9% (83.8 – 99.9)	99.3% (96.3 – 100)	50 % (37 – 63)	26.68%	0.18%	
[26] French Guiana	SD BIOLINE Dengue Duo NS1 Antigen	3,347	Retrospective/ blood	3,417 patients (52% of 6,521) at Cayenne hospital during the 2013 dengue epidemic in French Guiana were diagnosed with probable dengue by WHO 2009 criteria, with blood samples collected from febrile cases and nucleic acid testing for those with warning signs; incomplete cases were excluded.	RT-PCR	87% (80–93%)	92% (87–97%)	98.7%	7.7%	10.9%	0.14%	When considering only NS1 antigen results and not IgM, this RDT could be a suitable solution for diagnosing acute dengue infection in the early phase of the disease in healthcare centers where no laboratory services are available, in the early phase of the disease.
[27] Taiwan	SD BIOLINE Dengue Duo NS1 Ag + Ab Combo assay	8,989,	Retrospective/ serum	Serum samples from patients with suspected dengue were collected at Clinical Virology Laboratory of National Cheng Kung University Hospital between July and November 2015. patients were classified as mild, severe, or fatal dengue based on WHO 2009 criteria, with severe cases defined by plasma leakage, severe bleeding, or organ involvement.	qRT-PCR	89.4%	100%	0%	0%	0%	0%	These data indicate that NS1 antigen detection could be used for rapid diagnostic screening during large outbreaks.
	SD BIOLINE Dengue Duo IgM and IgG	8,954,				84.7%	100%	0%	0%	0%	0%	
[28] Thailand	Dengue-NS1- PAD	250	Retrospective cross-sectional/ serum	250 archived sera from dengue-suspected patients collected during the July–September 2019 epidemic at Phramongkutklao Hospital, Thailand.	Nested-PCR	88.89% (81.19–93.68)	86.67% (79.44–91.62)	84.62% (76.46 – 90.3)	90.43% (83.68 – 94.57)	6.67% (5.882 – 7.556)	0.13% (0.107 – 0.1537)	Dengue-NS1-PAD is a valuable tool for diagnosing DENV infections, especially for diagnosed patients with early acute phase samples with high viral load. Dengue-NS1-PAD has better sensitivity than SD-NS1 but less specific
	SD BIOLINE for NS1 RDT					87.88% (80.0– 92.93)	90.00% (83.33–94.19)	87.88% (80 – 92.93)	90% (83.33 – 94.19)	8.79% (7.44 – 10.38)	0.13% (0.11 – 0.16)	
[30] USA	SD BIOLINE Dengue RDT IgG/IgM	93	Cohort/ blood and serum	Two cohorts were studied: 24 adults in Oregon with past DENV/ZIKV infection and 69 participants in Ecuador post–2018 DENV peak.	RT-PCR	55%	≥98%	100%	87%	27.5%	0.46%	Overall, when tests were evaluated visually, sensitivity increased with time at the expense of specificity, limiting the reading of the RDT to earlier time points. Quantitative evaluation enabled the tests to reach recommended specificity and PPV, while also improving sensitivity and NPV.
[31] Thailand	IgM antibodies (Merlin)	259	Retrospective/ blood, plasma & serum	Patient samples from the Ragama Fever Study (Sri Lanka, 2006–2007) were collected from 259 febrile adults (median age 30; 69.5% male) at admission, discharge, and ~2 weeks later, with a median fever duration of 5 days before admission and a 16-day interval between paired samples.	PCR	72.7% (62.9–81.2)	73.8% (66.2–80.4)	63.2% (53.6–72.0)	81.4% (74.1–87.4)	2.72%	0.37%	This study provides strong evidence of the value of combining dengue antigen- and antibody-based test results in the RDT format for the acute diagnosis of dengue.
	IgM antibodies (Biosynex)					79.8% (70.5–87.2)	46.3% (38.3–54.3)	49.9% (40.1–55.8)	78.7% (69.1–86.5)	1.49%	0.44%	
	IgM antibodies (Standard Diagnostics)					79.2% (70.5–87.2)	89.4% (83.5–93.7)	82.3% (73.2–89.3)	87.7% (81.7–92.3)	7.47%	0.23%	
	IgM antibodies (Panbio)					70.7% (60.7–79.4)	80.0% (73.0–85.9)	68.6% (58.7–77.5)	81.5% (74.6–87.3)	3.54%	0.37%	
	NS1 antigen (Standard Diagnostics)					48.5% (38.5–58.7)	99.4% (96.6–100)	98.0% (89.1–100)	75.7% (69.3–81.4)	80.83%	0.52%	
	NS1 antigen (Bio-Rad)					58.6% (48.2–68.4)	98.8% (95.6–99.9)	96.7% (88.5–99.6)	79.4% (73.1–84.8)	48.83%	0.42%	
	NS1 antigen (Panbio)					58.6% (48.2–68.4) 92.5	92.5% (87.3–96.1)	82.9% (72.0–90.8)	78.3% (71.7–84.0	7.8%	0.45%	
	IgM antibodies and NS1 antigen (Standard Diagnostics)					92.9% (83.9–97.1)	88.8% (82.8–93.2)	83.6% (75.4–90.0)	95.4% (90.6–98.1)	8.29%	1.01%	
	IgM antibodies and NS1 antigen (Panbio)					89.9% (82.2–95.0)	75.0% (67.6–81.5)	69.0% (60.3–76.8)	92.3% (86.3–96.2)	3.60%	0.13%	
[32] Myanmar	NS1 antigen only (careUS Dengue Combo Kit)	202	A Hospital and Laboratory-based descriptive study/ serum	Clinically diagnosed dengue patients admitted to Mandalay Children Hospital (550 beds) in Myanmar provided single serum samples during the acute phase (≤7 days after fever onset) at admission, between July and August 2018.	RT-PCR	72.1% (63.9–79.4)	87.1% (76.1–94.3)	92.7% (86.0 –96.8)	58.1% (47.4 – 68.2)	5.6% (2.9–10.8	0.3% (0.2–0.4)	This study explored the evidence of the usefulness of RDT Kits at the point-of-care setting for the diagnosis of acute dengue infection. For these three commercially available RDT Kits, careUS Dengue Combo Kit was better than the other two. Combined detection of NS1 antigen, IgM, and IgG using RDT kits for diagnosis of dengue infection could be used by clinicians for getting an early diagnosis and effective treatment of the disease. It would be helpful for the diagnosis of primary and secondary DENV infection at the point-of-care setting.
	NS1 antigen only (Humasis Dengue Combo Kit)					68.6% (60.2–76.1)	90.3% (80.1–96.4)	94.1% (87.6 –97.8)	56.0% (45.7 –65.9)	7.7% (3.29–15.2	0.3% (0.2–0.5)	
	NS1 antigen only (Wondfo Dengue Combo Kit)					67.1% (58.7–74.8)	91.9% (82.2–97.3)	94.9% (88.6 –98.3)	55.3% (45.2 – 65.1)	8.3% (3.5–10.4)	0.3% (0.2–0.5)	
	IgM antibody only (careUS Dengue Combo Kit)					67.1% (58.7–74.8)	83.9% (72.3 – 92.0)	90.4% (83.0 – 95.3)	53.1% (42.7 – 63.2)	4.2% (2.3–7.4)	0.4% (0.3–0.5)	
	IgM antibody only (Humasis Dengue Combo Kit)					13.6% (8.4 20.4)	98.4% (91.3 – 99.9)	95.0% (75.1 – 99.9)	33.5% (26.7 – 40.9)	8.4% (1.2–11.1)	0.8% (0.8–0.9)	
	IgM antibody only (Wondfo Dengue Combo Kit)					19.3% (13.1–26.8)	95.2% (86.5 – 98.9)	90.0% (73.5 –97.9)	34.3% (27.2 – 41.9)	4.0% (1.3–12.6)	0.8% (0.7–0.9)	
	Combined NS1 antigen and IgM antibody (careUS Dengue Combo Kit)					92.1% (86.4–96.0)	75.8% (63.3 –85.8)	89.6% (83.4–94.0)	81.0% (68.6 – 90.3)	3.8% (2.4–5.9)	0.1% (0.1–0.2)	
	Combined NS1 antigen and IgM antibody (Humasis Dengue Combo Ki)					74.3% (66.2–88.2)	88.7% (78.1– 95.3)	93.7% (87.4–97.4)	60.4% (49.6 – 70.5)	6.6% (3.3–13.3)	0.3% (0.2–0.4)	
	Combined NS1 antigen and IgM antibody (Wondfo Dengue Combo Kit)					70.0% (61.7–77.4)	91.9% (82.2 –97.3)	95.1% (89.0 –98.4)	57.6% (47.2 –67.5)	8.7% (3.7–20.3)	0.3% (0.3–0.4)	
[33] Laos Vientiane	SD Bioline Dengue Duo RDT (Serum on RDT)	99	Retrospective/ Serum and blood	Samples were collected at two hospitals in Laos: Mahosot (Vientiane, urban) and Salavan (southern, rural, 679 km away). At Mahosot, 99 dengue-suspected patients (Aug–Nov 2013) were enrolled; at Salavan, 362 malaria-negative febrile patients (Jul–Oct 2012) were recruited. Venous and capillary blood were collected, with RDTs performed on whole blood and serum. Serum was stored at –80°C (Mahosot) and –20°C (Salavan), with dried blood/serum spots also preserved.	RT-PCR	85.4% (72.2–93.9)	98.0% (89.6–99.9)	97.6%	87.7%	42.7%	0.15%	There was 100% concordance between RDT and serum RT- PCR of infecting dengue serotype
	SD Bioline Dengue Duo RDT (Whole blood on RDT)					70.8% (55.9–83.0)	94.1% (83.8–98.8)	91.9%	77.4%	12%	0.31%	
Salavan	SD Bioline Dengue Duo (Serum on RDT)	362				94.0% (87.4–97.8)	93.9% (90.3–96.5)	85.45%	97.6%	15.41%	0.06%	
	SD Bioline Dengue Duo (Whole blood on RDT).					92.0% (84.8–96.5)	91.2% (87.1–94.4)	80%	96.76%	10.45%	0.09%	
[34] Taiwan	AsiaGen Dengue NS1 RDTs	122	Retrospective/ serum	A total of 122 serum samples (97 from dengue fever patients and 25 from healthy donors) were collected at Kaohsiung Medical University Hospital, stored at −80 °C, and tested by qRT-PCR and serotype-specific RT-PCR between 2015 and 2016.	RT-PCR	96.9%	100%	100%	89.30%	0%	0.03%	The results suggested that the two DENV-NS1 RDTs used in this study were promising for the timely diagnosis of DENV infection during dengue outbreaks, at least for DENV–2 in areas where authorized medical laboratories are not available or medical resources are limited
	SD BIOLINE Dengue NS1 RDT					100%	100%	100%	100%	0%	0%	
[35] Puerto Rico	SD BIOLINE Dengue Duo Anti-DENV IgM RDT	1678	Retrospective/ Serum	The CDC Dengue Branch (CDC-DB) supported health officials in investigating suspected dengue outbreaks in the Marshall Islands (2011–2012), Yap Island, FSM (2011), Angola (2013), and Fiji (2014), each characterized by a predominant DENV serotype for retrospective serotype-specific analysis. Suspected cases were defined as patients presenting with acute febrile illness, from whom serum specimens, along with demographic and clinical data, were collected at initial presentation; no convalescent samples were obtained.	RT-PCR	55.3–91.7% (38.3 – 71.4 to 61.5 – 99.8)	85.3– 98.5% (68.9 –95.1 to 96.2– 99.6)	-	-	3.76% to 61.13%	0.52 to 0.08%	This study design determined that the RDTs were a rapid method to confirming a dengue case in resource-limited regions and allowed for a rapid, more-focused outbreak response, including prevention methods such as community outreach, mosquito prevention/control, and clinician awareness.
	SD BIOLINE Dengue Duo Anti-DENV NS1 RDT					49.7 to 92.9% (42 – 57.4 to 76.5 – 99.1)	22.2 to 89.0% (6.4 – 47.6 to 84.2 – 92.7)			0.64% to 8.45%	2.27% to 0.08%	
**TT RESULTS**												
[12] Peru.	TT	Active = 1,095 or	Surveillance and cohort/serum	Between 2002 and 2011, dengue surveillance in Iquitos included passive monitoring at 13 clinics for febrile patients without other infection sources, with serum collected at acute and follow-up visits. In addition, two active cohorts (~4,500 and ~6,000 participants) were regularly visited at home to identify dengue-like illness, collect acute and convalescent serum samples, and monitor symptoms until recovery for seroconversion analysis.	PCR	52%	58%	45%	64%	1.24%	0.83%	It was demonstrated that the TT was more sensitive identifying dengue disease in women and those of younger age and that sensitivity increased the later a person came to a medical clinic for care.
		Passive = 12,453				56%	68%	55%	69%	1.75%	0.64%	
[14] Thailand	TT	176	Descriptive cross-sectional/ blood	Participants were patients admitted with suspected or provisional dengue infection, whose parents or guardians provided informed consent. Each underwent detailed history-taking, clinical examination, and blood sampling on admission for CBC, liver function, and dengue confirmation by ELISA and PCR at AFRIMS, with repeat testing on a second specimen collected 10–14 days later.		-	-	-	-	-	-	TT should be considered a simple clinical tool in assisting the diagnosis of dengue fever and other dengue infections and this can be combined with other clinical signs like fever and leucopenia.
[36] Puerto Rico	TT	284	Retrospective/ blood	Study participants were children and adults presenting with acute febrile illness (AFI) to the emergency department of Saint Luke’s Episcopal Hospital in Ponce, Puerto Rico, beginning in 2009. Eligible patients had a documented fever ≥38 °C or a history of fever lasting 2–7 days without an identifiable source, while those with defined infections such as otitis media, pneumonia, or pyelonephritis were excluded.	PCR	51.6% (33–69)	82.4 (76–87)	29.6 (18–43)	92.2 (87–95)	2.9%	0.59%	This study indicates that a combination of two rapid, widely available tests can assist clinicians in distinguishing dengue from other illnesses with similar signs and symptoms.
[37] Taiwan.	TT	581	Retrospective/ serum	At National Cheng Kung University Hospital (Jan–Dec 2007), patients were enrolled with clinically suspected dengue, defined by fever ≥38°C for under 7 days plus at least two typical symptoms or physician suspicion. Pediatric cases were those under 18 years. Patients were categorized as laboratory-positive (confirmed by IgM/IgG serology or RT-PCR), laboratory-negative, or indeterminate (no convalescent specimen). Demographics, comorbidities, clinical features, and lab data were collected, with key findings including leukopenia, thrombocytopenia, elevated AST/ALT, prolonged aPTT, and low CRP.	RT-PCR	34.2%	100%	100%	28.2%	0.34%	0.66%	These clinical and laboratory results could be used as prognostic indicators to aid in the early detection of dengue infection in Taiwan.
[38] Brazil	TT	30, 760	Retrospective	Study participants were patients meeting Brazil’s Ministry of Health criteria for suspected dengue, defined as acute febrile illness lasting up to seven days with at least two symptoms such as headache, retro-orbital pain, myalgia, arthralgia, prostration, rash, bleeding, or epidemiological risk factors (residence/travel in endemic areas).	PCR	11.9 % (0.11 to 0.12)	88.9% (0.88 – 0.89)	31.6 % (0.31 to 0.32)	70.3 % (0.70 – 0.71)	1.08%	0.99%	Therefore, the TT was more effective in detecting cases that were truly negative than positive. These results suggest that the TT should not be used as a diagnosis of dengue, however, if it is necessary to use it, as is the case in very poor dengue-endemic areas where more sensitive and specific laboratory tests are not available, then it to be done with great caution for screening giving rise to suspicion of dengue cases.

**Abbreviations: CI**: Confident interval, **PPV:** Positive Predictive Value, **NPV:** Negative Predictive Value, **PLR:** Positive likelihood ratio, **NLR:** Negative likelihood ratio, **RDTs:** Rapid Diagnostic Tests, **NS1:** Non-Structural Protein 1, **PCR:** Polymerase Chain Reaction, **EIA:** Enzyme Immunoassay, **DENV-1:** Dengue Virus Serotype 1, **LAMP:** Loop-Mediated Isothermal Amplification, **SD:** Standard, **RT-PCR:** Reverse Transcription Polymerase Chain Reaction, **qRT-PCR:** Quantitative Reverse Transcription Polymerase Chain Reaction **IgG:** Immunoglobin G, **IgM:** Immunoglobin M, **DENV:** Dengue Virus, **mAbs:** Monoclonal Antibodies, **RDT:** Rapid Diagnostic Test, **PAD:** Paper-based Analytical Device, **USA:** United States of America, **DENV-2:** Dengue Virus Serotype 2, **TT:** Tourniquet Test, **ZIKV: Zika virus,**

### Study characteristics

Twenty-three (23) eligible studies were included (Table 1). These studies, conducted between 2001 and 2022, spanned 21 countries: Australia, Thailand, Vietnam, United States of America (USA), Netherlands, China, Canada, England, India, Japan, Germany, Switzerland, Republic of Korea, New Caledonia, Malaysia, Italy, Brazil, Puerto Rico, Peru, Taiwan, and French Guiana. The regional distribution of these studies is also detailed in Table 1.

Of the 21 countries, studies involving RDTs were conducted in 16 countries: Switzerland, New Caledonia, Germany, Japan, India, England, China, the Netherlands, Canada, the USA, Vietnam, Thailand, Australia, the Republic of Korea, Italy, and French Guiana. TT was studied in 5 countries: Taiwan, Thailand, Brazil, Peru, and Puerto Rico.

Peru (n = 13,548) and Brazil (n = 30,760) had the largest study populations, using TT and RDTs, respectively. Most of these studies focused on individuals with suspected acute DENV infection. The most frequently used RDTs were the Standard™ Dengue Duo test, which demonstrated the highest sensitivity among the assays, followed by the Panbio® Dengue RDT. In all studies, RT-PCR was used as the reference standard. Notably, one study involving RDTs reported issues of serological cross-reactivity.

### Risk of bias and study quality assessment

Each selected study was assessed using STARD [16] and QUADAS-2 [17] guidelines. The STARD tool assesses five domains, including background and objectives, methods, results, discussion, and registration, while QUADAS-2 evaluates four key areas of diagnostic study quality: patient selection, index test, reference standard, and flow and timing.

Due to the use of patient data from databases, potential bias from multiple assessors, or the inclusion of patients with pre-existing conditions, a high risk of bias in the patient selection domain was observed in five studies, while the other 18 studies were evaluated of a low risk of selection bias. Regarding PCR as a reference standard, all studies demonstrated a low risk of bias. For the domain of index, unclear risk of bias was observed in four studies due to insufficient reporting or methodological concerns.

**Figure 3.**
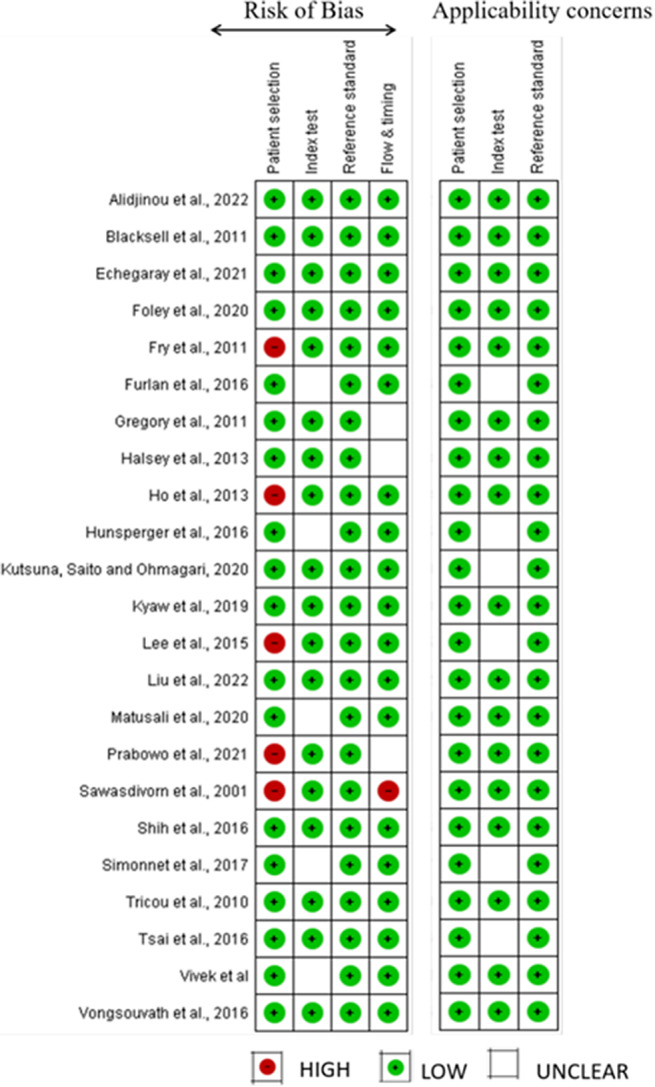
Quality Assessment of Diagnostic Accuracy Studies II.

Study quality assessments are illustrated in Figure 3. Only one study employed random sampling [28]. Based on the cumulative data, 100%, 56%, and 15% of participants underwent PCR, RDT, and TT, respectively.

## Discussion

A summary of all investigations, including the types of samples used and patient characteristics, is provided in Table 1. Across all studies, a total of 7,513 samples were evaluated using RDTs, with serum, plasma, and whole blood being the primary sample types. Only one study specifically assessed the performance of dengue RDTs for detecting remote prior infection [30], providing limited insight into the IgG component of RDTs in individuals with a history of DENV exposure.

Among the 18 RDT studies, four [22, 31, 32, 35] evaluated the IgM component, while fourteen [3, 19, 20, 22–25, 27–29, 31–32, 34–35] focused on NS1 antigen detection. Six studies [3, 22, 24, 25, 29, 30] assessed combined NS1/IgM detection, two [30, 32] evaluated IgM/IgG, and four [3, 22, 24, 27] assessed all three markers (IgG, IgM, NS1). This distribution reflects the focus on acute primary infections, in which IgG levels are often absent or minimal depending on the illness stage.

The mean sensitivity of RDTs detecting IgM alone was 61.4% (SD = 28.6), with a specificity of 80.3% (SD = 16.4). For NS1 alone, the mean sensitivity was 79.2% (SD = 13.7), and specificity was 94.6% (SD = 5.1). Combined detection of IgM and NS1 increased sensitivity to 80.4% (SD = 14.5) and specificity to 89.4% (SD = 11.3). Notably, combining all three biomarkers (IgG, IgM, and NS1) yielded the highest diagnostic performance, with a mean sensitivity of 90.98% (SD = 8.4) and specificity of 90.8% (SD = 14.6).

Table 1 provides detailed estimates for each RDT’s likelihood ratios, predictive values, and 95% confidence intervals. The majority of included studies were carried out in dengue-prone areas; they often lacked information on co-infection with other flaviviruses or prior treatment history. This limited the ability to account for potential serologic cross-reactivity, which may lead to false positives, especially in areas where viruses like Zika co-circulate. Only one study thoroughly assessed flaviviral cross-reactivity.

Most RDT studies used whole blood, suitable for point-of-care testing. One innovative study [33] demonstrated that nucleic acid amplification and DENV typing could be conducted directly from used RDTs, making field-based surveillance more feasible. Results showed strong concordance between NS1 RDT-derived samples and serum-based RT-PCR results, with agreement rates of 82.8% (Vietnam) and 91.4% (Malaysia) using blood, and 91.9% and 93.9%, respectively, using serum. There was 100% concordance in identifying the infecting serotype between the two methods.

Five RDT studies [20, 24, 25, 32, 34] successfully differentiated among all four dengue serotypes, with DENV-1 being the most prevalent. One study compared RDT performance across whole blood, serum, and plasma, as summarized in Table 1. However, due to inconsistent reporting on vaccination status, age, co-infections, and time since symptom onset, subgroup analyses were not possible. Overall, the mean sensitivity and specificity of RDTs were 76.2% (SD = 13.8) and 91.5% (SD = 10.3), respectively (Figure 3).

**Figure 4.**
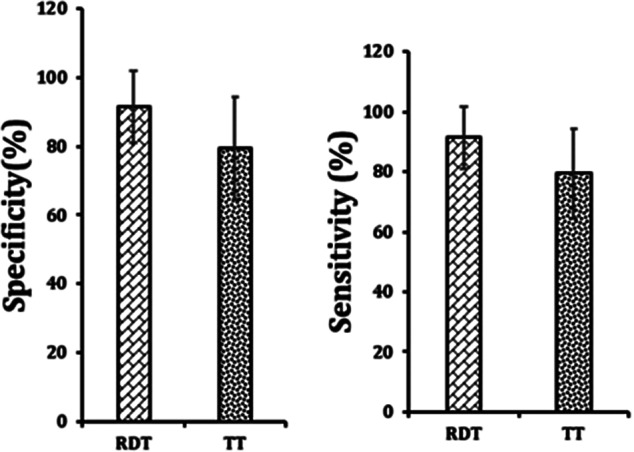
Graphical representation of mean sensitivity and specificity for both the tourniquet test (TT) and rapid diagnostic tests (RDTs).

Furthermore, five studies [12, 14, 36–38] evaluated the TT, including both DF and DHF cases. The mean sensitivity of TT was 48.6% (SD = 24.9), and specificity was 79.5% (SD = 14.9) (Figure 4), with 95% CI ranges varying. 0 to 0.9 was the range observed for positive predictive values (PPV) and 0 to 2.9 for negative predictive values (NPV). These findings suggest that TT is better at identifying true negatives than true positives. The positive likelihood ratio ranged from 0 to 2.9, suggesting limited ability to increase post-test probability of dengue in TT-positive individuals. Similarly, the negative likelihood ratio (0 to 0.99) showed that a negative TT result had limited discriminatory power. TT sensitivity was lowest on day 0 of illness onset and highest on day 7 and beyond, as reported by Halsey et al. [12]. Specificity remained stable across illness days. Sensitivity was higher in younger patients and females, whereas specificity was slightly higher in males. Repeat testing moderately improved sensitivity (to 60%) but reduced specificity (to 56%).

Using PCR as the diagnostic standard, TT demonstrated less diagnostic accuracy compared to RDTs. The TT’s mean sensitivity (48.6%) and specificity (79.5%) were significantly lower than RDTs (76.2% and 91.5%, respectively). However, two TT studies [14, 37] had a high risk of bias, potentially skewing sensitivity downward. A limitation of this analysis is that most TT studies used ELISA, not PCR, as the reference standard. Thus, fewer TT studies met the inclusion criteria. Additionally, the inconsistent reporting across primary studies prevented evaluation of TT’s effectiveness in specific subgroups. It also raises concerns about whether TT offers any real advantage over clinical evaluation alone. Further research using PCR as the gold standard is warranted to fully understand the diagnostic utility of TT. In particular, assessing TT performance by day of illness, gender, age, and dengue subtype could provide valuable insights for clinical application. For RDTs, larger studies are needed to explore the impact of pre-existing immunity and dengue subtype on diagnostic accuracy.

Finally, manufacturers of dengue RDTs should be encouraged to develop and validate new tests with improved sensitivity and specificity, incorporating novel antigen or antibody targets. Such improvements would significantly enhance dengue diagnosis, particularly in low-income countries where rapid, reliable, and affordable tools are urgently needed.

## Conclusion

In resource-constrained settings where advanced laboratory diagnostics are unavailable, it is essential to distinguish between the utility of the TT and RDT for dengue detection. Given the TT’s consistently low sensitivity, it should only be used alongside other diagnostic methods for dengue. If employed, particularly in severely resource-limited, dengue-endemic regions, it must be interpreted with caution and only in conjunction with clinical findings. In contrast, RDTs demonstrate superior diagnostic performance compared to TT, particularly when benchmarked against PCR. Therefore, RDTs should be prioritized over TT in the diagnostic approach to DF in low-resource environments.

## Supporting information

10.1017/S0950268825100460.sm001 Baje et al. supplementary material Baje et al. supplementary material

## Data Availability

Will be made available on request.

## Supplementary material

The supplementary material for this article can be found at http://doi.org/10.1017/S0950268825100460.
