# Supplementary material for: Dengue fever diagnosis in resource-limited settings
Source: Epidemiol Infect. 2025 Aug 22;153:e105. doi: 10.1017/S0950268825100460 (PMC12455512; doi:10.1017/S0950268825100460)
Supplement: Baje et al. supplementary material [file S0950268825100460sup001.docx]

**Supplementary Table1.** Search Strategy

| **Date** | **Database** | **Search string** | **Hits** |
| --- | --- | --- | --- |
| TIME 10:50am  DATE: 8^th^ April 2023 | PubMed | (((((((("tourniquet test"[Text Word] OR "capillary fragility test"[Text Word] OR "capillary fragility tests"[Text Word]) AND "PCR"[All Fields]) OR "multiplex polymerase chain reaction"[MeSH Terms]) AND "rapid diagnostic tests"[Text Word]) OR "rapid diagnostic test"[Text Word]) AND "dengue"[MeSH Terms]) OR "breakbone fever"[Text Word]) AND "diagnosis"[Text Word]) NOT (ELISA) | **37** |
| TIME 4:00pm  DATE: 9^th^ April 2023 | Scopus | ( TITLE-ABS-KEY ( ( comparison ) OR ( "diagnostic accuracy" ) OR ( tourniquet ) OR ( "rapid diagnostic test*" ) ) AND TITLE-ABS-KEY ( ( dengue ) OR ( dengue AND diagnosis ) ) AND TITLE-ABS-KEY ( ( pcr ) ) AND NOT TITLE-ABS-KEY ( elisa ) ) AND ( EXCLUDE ( SUBJAREA , "CHEM" ) OR EXCLUDE ( SUBJAREA , "AGRI" ) OR EXCLUDE ( SUBJAREA , "PHAR" ) OR EXCLUDE ( SUBJAREA , "ENGI" ) OR EXCLUDE ( SUBJAREA , "CENG" ) OR EXCLUDE ( SUBJAREA , "PHYS" ) OR EXCLUDE ( SUBJAREA , "ENVI" ) OR EXCLUDE ( SUBJAREA , "DENT" ) ) AND ( EXCLUDE ( DOCTYPE , "ch" ) OR EXCLUDE ( DOCTYPE , "le" ) OR EXCLUDE ( DOCTYPE , "re" ) ) AND ( EXCLUDE ( LANGUAGE , "French" ) OR EXCLUDE ( LANGUAGE , "Portuguese" ) OR EXCLUDE ( LANGUAGE , "Spanish" ) OR EXCLUDE ( LANGUAGE , "Chinese" ) ) AND ( EXCLUDE ( EXACTKEYWORD , "Chikungunya" ) OR EXCLUDE ( EXACTKEYWORD , "Chikungunya Virus" ) OR EXCLUDE ( EXACTKEYWORD , "Zika Virus" ) OR EXCLUDE ( EXACTKEYWORD , "Malaria" ) OR EXCLUDE ( EXACTKEYWORD , "Zika Fever" ) OR EXCLUDE ( EXACTKEYWORD , "Zika Virus Infection" ) OR EXCLUDE ( EXACTKEYWORD , "Japanese Encephalitis Virus" ) OR EXCLUDE ( EXACTKEYWORD , "Yellow Fever Virus" ) ) | 140 |
| TIME 5:20pm DATE:9th April 2023 | Science Direct | (comparison OR "diagnostic accuracy" OR tourniquet OR "rapid diagnostic test" ) ) AND ( ( dengue OR dengue AND diagnosis ) AND ( ( pcr ) ) NOT ( elisa ) ) | 498 |
